# Three-Dimensional Object Recognition and Registration for Robotic Grasping Systems Using a Modified Viewpoint Feature Histogram

**DOI:** 10.3390/s16111969

**Published:** 2016-11-23

**Authors:** Chin-Sheng Chen, Po-Chun Chen, Chih-Ming Hsu

**Affiliations:** 1 Graduate Institute of Automation Technology, National Taipei University of Technology, Taipei 106, Taiwan; saint@ntut.edu.tw (C.-S.C.); t103618036@ntut.org.tw (P.-C.C.); 2 Department of Mechanical Engineering, National Taipei University of Technology, Taipei 106, Taiwan

**Keywords:** vision-guided robot, Kinect sensor, viewpoint feature histogram descriptor, iterative closest point

## Abstract

This paper presents a novel 3D feature descriptor for object recognition and to identify poses when there are six-degrees-of-freedom for mobile manipulation and grasping applications. Firstly, a Microsoft Kinect sensor is used to capture 3D point cloud data. A viewpoint feature histogram (VFH) descriptor for the 3D point cloud data then encodes the geometry and viewpoint, so an object can be simultaneously recognized and registered in a stable pose and the information is stored in a database. The VFH is robust to a large degree of surface noise and missing depth information so it is reliable for stereo data. However, the pose estimation for an object fails when the object is placed symmetrically to the viewpoint. To overcome this problem, this study proposes a modified viewpoint feature histogram (MVFH) descriptor that consists of two parts: a surface shape component that comprises an extended fast point feature histogram and an extended viewpoint direction component. The MVFH descriptor characterizes an object’s pose and enhances the system’s ability to identify objects with mirrored poses. Finally, the refined pose is further estimated using an iterative closest point when the object has been recognized and the pose roughly estimated by the MVFH descriptor and it has been registered on a database. The estimation results demonstrate that the MVFH feature descriptor allows more accurate pose estimation. The experiments also show that the proposed method can be applied in vision-guided robotic grasping systems.

## 1. Introduction

Robotic grasping systems cannot quickly or accurately recognize randomly oriented objects that exit an assembly line or which are located on an assembly table so machine vision is used to solve this problem. Previous studies have proposed efficient algorithms for object recognition and pose estimation [1,2,3]. However, these algorithms are not suitable for use in household environments and industrial scenarios [4,5] because the speed of calculation does not satisfy user requirements in these settings. Household environments are unstructured and contain diverse object types, so there are high calculation costs. However, in industrial applications environments can be properly designed, which is why machine vision is widely used in industrial scenarios. The development of 3D image processing [6,7] is not as mature as 2D image processing [8], but the use of 3D image processing for 3D object recognition and registration is possible. The use of 3D object recognition has several advantages. The range images provide depth information, which helps resolve any ambiguity caused by perspective projection in 2D vision. Moreover, for some technologies, such as time-of-flight cameras, the features extracted from range images are unaffected by illumination. However, 3D-based object recognition still imposes additional challenges related to scaling, viewpoint variation, partial occlusions, and background clutter. The viewpoint feature histogram (VFH) [1] descriptor is a novel 3D feature descriptor for object recognition and identifying the pose in mobile manipulation and grasping applications where there are six-degrees-of-freedom (6-DOF). The VFH descriptor is robust against a large degree of surface noise and missing depth information so it is reliable for stereo data. However, object pose estimation fails when objects are symmetrically placed with relation to the viewpoint. To overcome this problem, a modified viewpoint feature histogram (MVFH) descriptor is proposed that consists of two parts: a surface shape component that comprises an extended fast point feature histogram (FPFH) and an extended viewpoint direction component. The MVFH descriptor characterizes an object’s pose and increases the ability to identify objects with mirrored poses. The key contribution of this paper is the design of a novel, accurate, and computationally efficient 3D feature that allows object recognition and the identification of the pose for a vision-guided robotic (VGR) grasping system when there are 6-DOF.

The structure of this paper is as follows: the system architecture is described in Section 2. The object recognition and registration algorithm is discussed in Section 3. Section 4 describes the experimental setup and the resulting computational and recognition performance. Finally, conclusions and suggestions for future research are given in Section 5.

## 2. System Architecture

### 2.1. Hardware Setup

**Figure 1:**
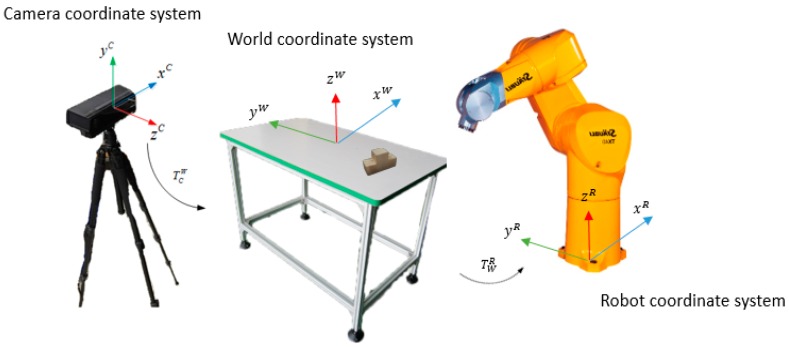
Hardware configuration.

Figure 1 illustrates the architecture of the hardware system, which comprises three parts: a 3D sensor (Microsoft Kinect), a robotic arm with 6-DOF and the working table. The Kinect sensor captures 3D point cloud data, the robot is guided to grasp objects and the working table simulates an assembly table.

### 2.2. Algorithm for the Robotic Grasping System

**Figure 2:**
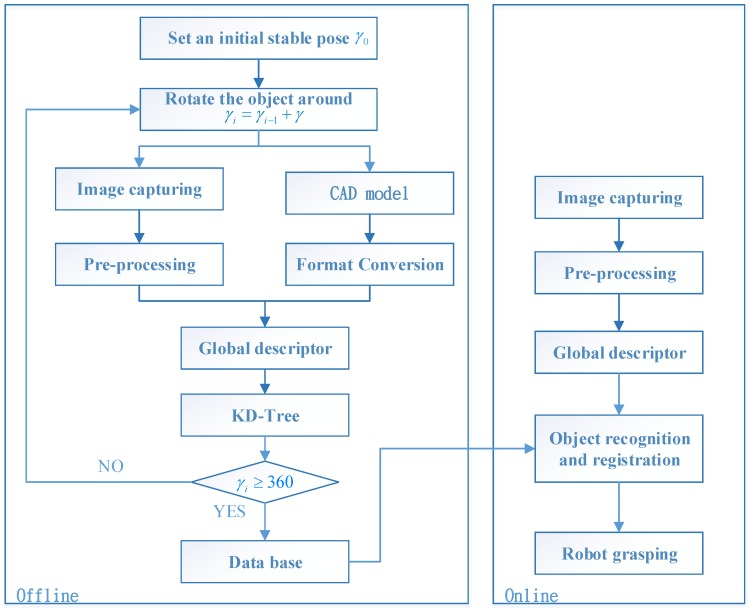
The architecture for the proposed algorithm.

The software system involves offline and online phases, as shown in Figure 2. In the offline phase, a complete pose database for an object is established. When the different stable poses of an object have been confirmed, the robot must be taught how to grasp the object from each stable pose. Information related to the object is then saved in a database that contains 3D data, grasping postures, descriptor histograms and classifications for each stable pose. In the online phase, the 3D sensor captures point cloud data. With reference to the database, the closest sample is found by comparing the histogram for a descriptor. Finally, the refined pose is registered, using iterative closest point (ICP) pose refinement and the robot is then guided to grasp the object.

### 2.3. Database for Object Recognition and Registration

**Figure 3:**
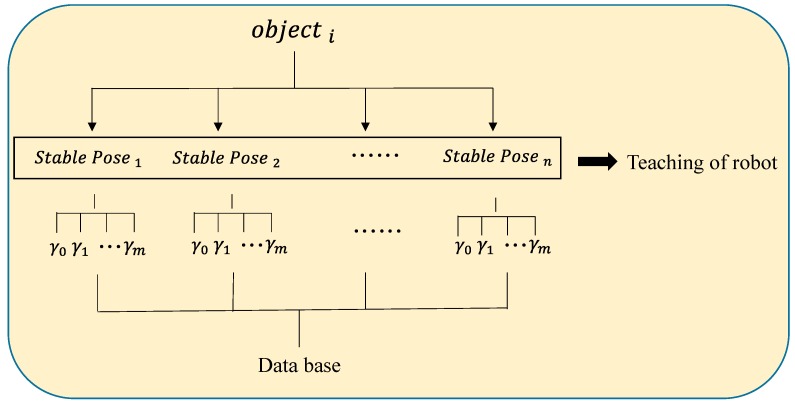
Database of stable positions.

An object that is placed on the table usually has more than one stable pose. Using one grasping posture to grasp an object in any situation is impossible because the environment has restrictions and the robot has a limited working area. Therefore, a robot must be taught how to grasp objects in any stable pose. When the initial posture is determined, the final posture is obtained by rotating the view angle for an object and saving the image data and feature description in a database. The database for stable positions for the proposed method is shown in Figure 3.

## 3. Object Recognition and Registration

### 3.1. Pre-Processing

**Figure 4:**
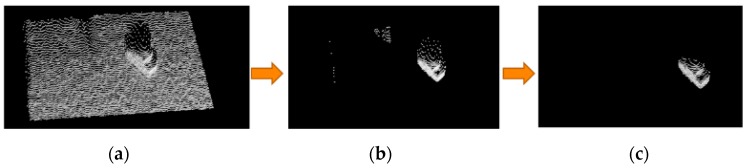
Results for (**a**) ROI isolation; (**b**) plane segmentation and (**c**) statistical outlier removal.

When the image data is being captured, the data are transformed into a global coordinate system by using a previously reported camera calibration method [9] to facilitate subsequent processing. The Kinect sensor outputs a considerable volume of point cloud data, but only some of this data is relevant to a specific object. Two steps are used to filter unnecessary data: plane segmentation and statistical outlier removal. Firstly, the image is segmented, to isolate the region of interest (ROI), as shown in Figure 4a since we have the (x, y, z) position of each point in the point cloud, a simple pass through filter [10] that allows only points satisfying the predefined conditions was deployed. As can be seen from the Figure 4a, only the target object for recognition is left. After the ROI isolation operation, a RANSAC-based plane segmentation [11] is used to identify the supporting plane in the point cloud. Then the inliers on the plane are segmented out and the resulted point cloud is shown in Figure 4b. Next, an outlier removal algorithm relying on the statistical characteristic is adopted [12]. For each point, we compute the mean distance from it to all its nearest k neighbors. All points whose mean distances are outside a defined interval can be considered as outliers. Finally, statistical outliers are removed, to reduce noise, as shown in Figure 4c. Another pre-processing step that significantly reduces the computation time is the down-sampling step. The raw point cloud captured by Kinect is too dense. We set the voxel size to 0.3 cm cube for the resolution of down-sampling operation [13], which means that points within the 0.3 cm cube will be averaged and represented by the centroid of these points.

### 3.2. Gobal Descriptor Estimation

**Figure 5:**
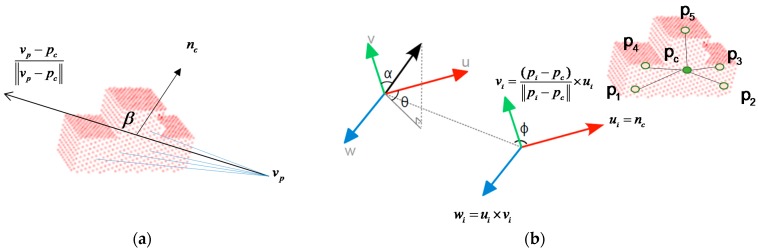
Object description: (**a**) viewpoint feature and (**b**) extended FPFH.

The global descriptors for an object are high-dimensional representations of the object’s geometry and are engineered for the purposes of object recognition, geometric categorization and shape retrieval. The VFH [1] is a novel representation of point cloud data for problems that are related to object recognition and pose estimation when there are 6-DOF. The VFH is a compound histogram that comprises the view feature and extended FPFH, as shown in Figure 5. It represents four angular distributions for surface normals. Let $p_{c}$ and $p_{i}$ denote the cloud gravity center and any point belonging to the cloud, and $n_{c}$ and $n_{i}$ denote the vector with initial point at $p_{c}$ and coordinates equal to the average of all surface normals and the surface normal estimated at point $p_{i}$. The term $\left(u_{i},v_{i},w_{i}\right)$ is defined as follows:

(1) $$u_{i}=n_{c},$$

(2) $$v_{i}=\frac{\left(p_{i}-p_{c}\right)}{\left\|p_{i}-p_{c}\right\|}\times u_{i},\text{ and}$$

(3) $$w_{i}=u_{i}\times v_{i}.$$

The normal angular deviations $\cos(\alpha_{i})$, $\cos(\beta_{i})$, $\cos(\phi_{i})$ and $\theta_{i}$ for each point $p_{i}$ and its normal $n_{i}$ are given by:

(4) $$cos(\alpha_{i})=v_{i}\cdot n_{i},$$

(5) $$cos\left(\beta_{i}\right)=n_{i}\cdot (\frac{v_{p}-p_{c}}{\|v_{p}-p_{c}\|}),$$

(6) $$cos\left(\phi_{i}\right)=n_{i}\cdot (\frac{p_{i}-p_{c}}{\|p_{i}-p_{c}\|}),\text{ and}$$

(7) $$\theta_{i}=tan^{-1}(\frac{w_{i}\cdot n_{i}}{u_{i}\cdot n_{i}}).$$

**Figure 6:**
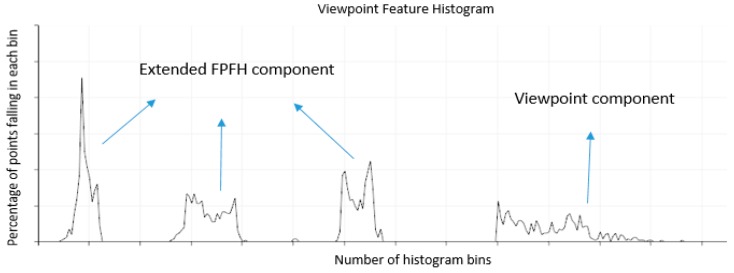
Example of the resultant VFH for an object.

For the extended FPFH, each angle requires 45 bins, and the VFH requires 128 bins. Finally, the histogram uses a total of 263 bins to describe the image. Figure 6 shows the VFH for an object.

**Figure 7:**
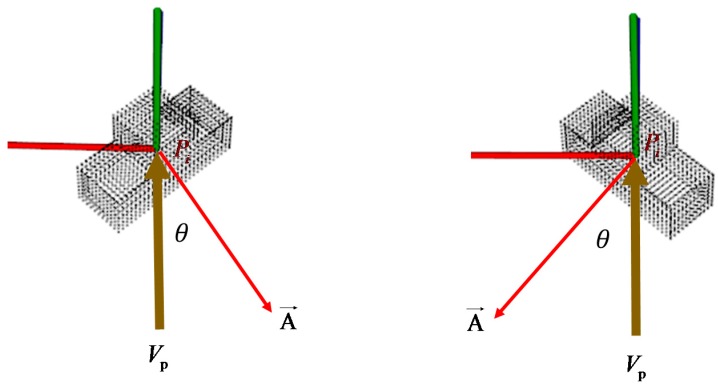
Poses that are symmetrical along the viewing direction.

Rusu et al. [1] presented the VFH as a novel 3D feature descriptor for object recognition and identification of the pose in mobile manipulation and grasping applications where there are 6-DOF. However, several accurate 3D pose estimation limitations were encountered. For example, all of the surfaces of a specified object might be flat, so their VFHs can generate false-positive results in some symmetric poses, as shown in Figure 7. The vector $\vec{A}$ represents a surface normal estimated at point $p_{i}$. Although these two poses are mirrored along the *x*-axis, the two VFHs are highly similar because their shapes are similar or identical. In this situation, the object is correctly recognized by the VFH, but these poses cannot be appropriately identified.

### 3.3. MVFH

To solve this problem, the specific VFH descriptor must be modified. The main reason that the two poses that are mirrored along the *x*-axis give two similar VFHs is that the viewpoint direction component in the VFH cannot be identified in mirrored cases. To overcome this problem, an MVFH descriptor is proposed and detailed as follows.

To increase a system’s ability to identify objects with mirrored poses, the viewpoint direction component in the VFH is given three components. These components measure the relative pan, tilt and yaw angles between the viewpoint direction at the central point and each surface normal. MVFHs represent three angular distributions of a surface normal. Let $v_{P}$ denotes the view direction vector for a given object in the partial view of the camera coordinate system. The extended viewpoint components $\left(U_{i},V_{i},W_{i}\right)$ are defined as:

(8) $$U_{i}=n_{c},$$

(9) $$V_{i}=(v_{P}-p_{i})\times U_{i},\text{ and}$$

(10) $$W_{i}=U_{i}\times V_{i}.$$

The normal angular deviations $cos(\alpha_{i}^{M})$, $cos\left(\phi_{i}^{M}\right)$, and $\theta_{i}^{M}$ for each point $p_{i}$ and its normal $N_{i}$ are given by:

(11) $$cos(\alpha_{i}^{M})=V_{i}\cdot N_{i},$$

(12) $$cos\left(\phi_{i}^{M}\right)=N_{i}\cdot (\frac{p_{i}-p_{c}}{\|p_{i}-p_{c}\|}),\text{ and}$$

(13) $$\theta_{i}^{M}=tan^{-1}(\frac{W_{i}\cdot N_{i}}{U_{i}\cdot N_{i}}).$$

**Figure 8:**
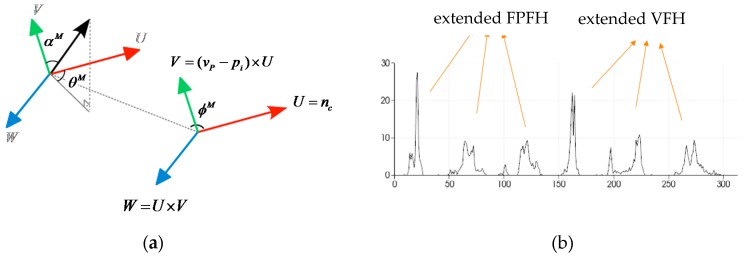
MVFH description: (**a**) viewpoint feature and (**b**) example of the resultant MVFH for one object.

**Figure 9:**
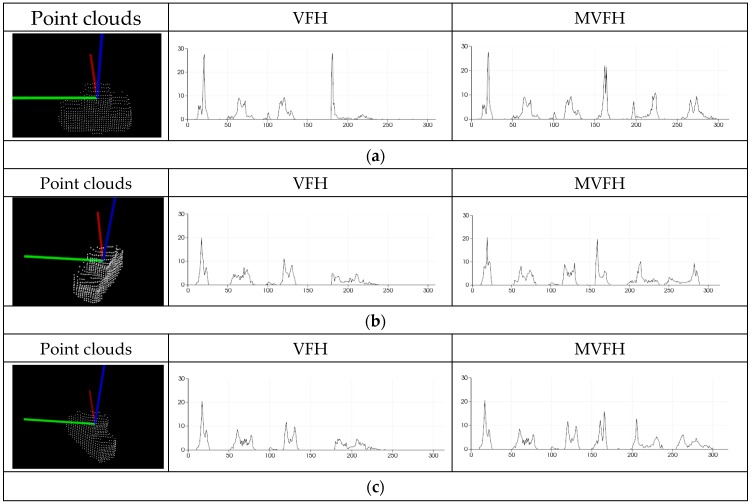
Comparison of two descriptors (VFH and MVFH) for three poses: (**a**) when the normal direction of the object surface identical to the viewpoint direction; (**b**) when there is a yaw angle of +30° and (**c**) when there is a yaw angle of −30°.

The default MVFH implementation uses 45 binning subdivisions for each of the three extended FPFH values and another 165 binning subdivisions for the extended viewpoint components, which results in a 300-byte array of float values. The new assembled feature is therefore called the MVFH. Figure 8 shows this concept with the new feature, which consists of two parts: a surface shape component that comprises an extended FPFH and an extended viewpoint direction component. Figure 9 shows the ability to identify objects with mirrored poses when the VFH and MVFH are used. Figure 9a shows a case in which the normal direction of the object surface is identical to the viewpoint direction. In the VFH and MVFH in Figure 9a, the MVFH contains more viewpoint direction components than the VFH. Figure 9b,c respectively show cases with yaw angles of +30° and −30°from the normal direction of an object surface in the viewpoint direction. These two viewpoint direction components of the VFH in Figure 9b,c could result in a false pose recognition for object grasping because these two VFH descriptors have similar matching scores at the registraton stage. However, the two viewpoint direction components of the MVFH descriptors in Figure 9b,c are significantly different in cases of mirrored object poses.

**Figure 10:**
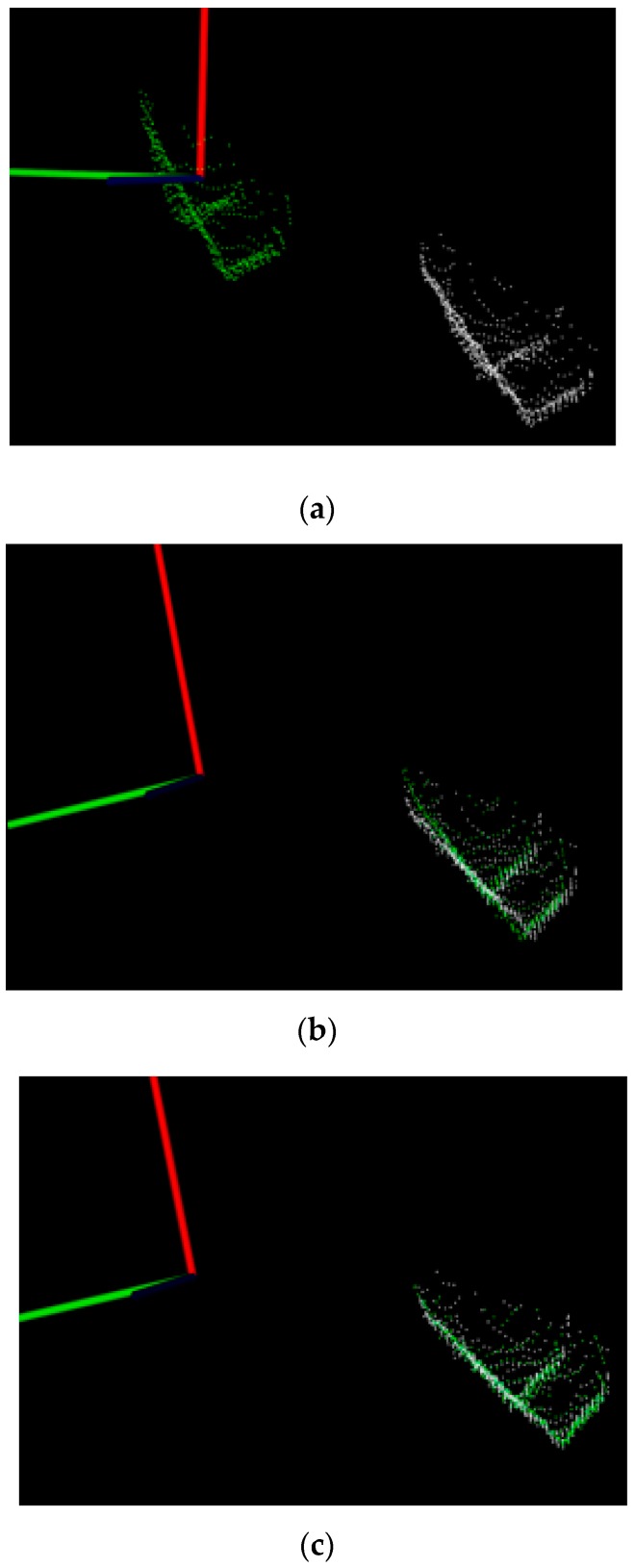
Object recognition and registration results: (**a**) recognition using the MVFH in the scan (color: white) and database (color: green) point clouds; (**b**) shifting procedure and (**c**) pose refinement using ICP.

Here, an object is first scanned using Kinect 2; the collected cloud (white) is shown in Figure 10a. For object registration, the collected cloud is compared with the poses stored in the database by using MVFHs and the nearest neighbor classifier [14]. The selected winning pose (green) is shown in Figure 10a. The selected winning pose is then moved to the centroid position of the recognized object (white), as shown in Figure 10b. After object recognition and rough pose estimation with the MVFH, the ICP algorithm [15] is used to minimize the difference between two groups of points to refine the estimated 6-DOF pose. The algorithm iteratively revises the transformation to minimize the distance from the reference (green) to the scaned point cloud until the specified number of iterations is reached or the distance error is below a threshold. Finally, the Figure 10c shows the object pose refinement after ICP process.

## 4. Experimental Results

**Figure 11:**
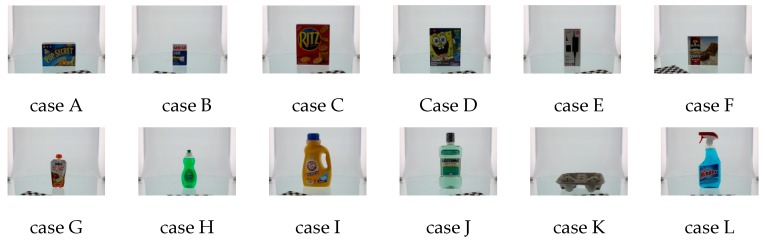
Twelve tested cases [16].

**Figure 12:**
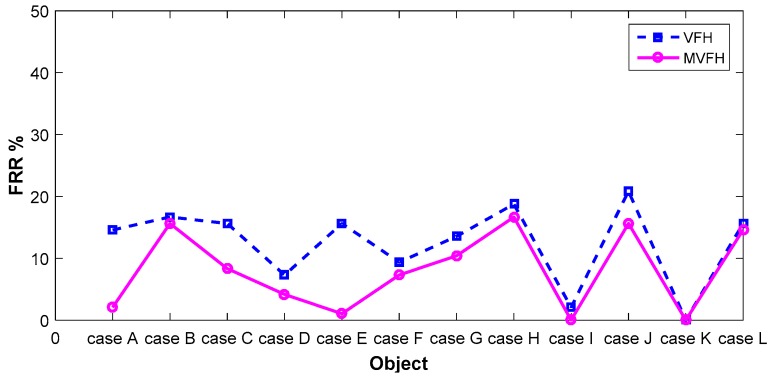
False recognition rate for the mirrored poses.

**Figure 13:**
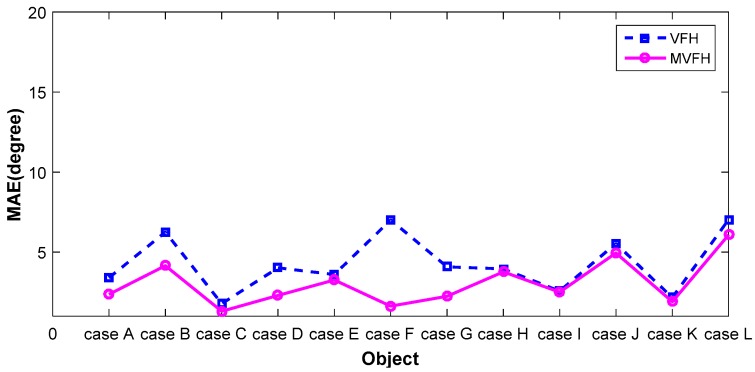
The pose estimation performance.

To demonstrate improvement in pose retrieval by using the proposed feature (MVFH) versus the VFH feature, available data sets [16] on the Internet (e.g., http://rll.berkeley.edu/bigbird/) were used to test our proposed method. The data sets provide 600 point clouds by taking shots from five polar angles and 120 azimuthal angles, with the azimuthal angles equally spaced by 3°. To demonstrate improvement in pose retrieval using the proposed feature (MVFH) versus the VFH feature, twelve tested cases shown in Figure 11 were chosen from the BIGBIRD data set. Each tested case has 24 point clouds, which was derived by taking 24 azimuthal angles equally spaced with 15° to establish a complete pose database. Ninety-six point clouds were tested to characterize an object’s pose and enhance the system’s ability to identify objects with mirrored poses in each case. Figure 12 shows false recognition rate (FRR) for the mirrored poses. For the pose estimation, one measure [mean absolute error, (MAE)] of pose estimation was used to validate the performance of using the VFH descriptor. Overall (totally 12 × 96 = 1152 pose), the proposed feature (MVFH) has higher accuracy than the VFH descriptor, as shown in Figure 13. For the FRR of the mirrored poses, the overall FRR of the MVFH descriptor (7.98%) was less than that of the VFH descriptor (12.54%). Table 1 presents the computation time results, which were required for the test cases. When the object was recognized and the corresponding rough pose was estimated using the VFH and the pose was further refined using ICP, the average computation time was 0.25634 s. The MVFH was also used to avoid false pose recognition, reducing the average computation time to 0.22179 s. From Figure 12 and Figure 13 and Table 1, the MVFH has overall higher pose estimation accuracy than the VFH descriptor under the same computation time. The experimental results show that the proposed method can improve pose retrieval performance by using the proposed feature (MVFH) versus the VFH feature.

**Figure 14:**
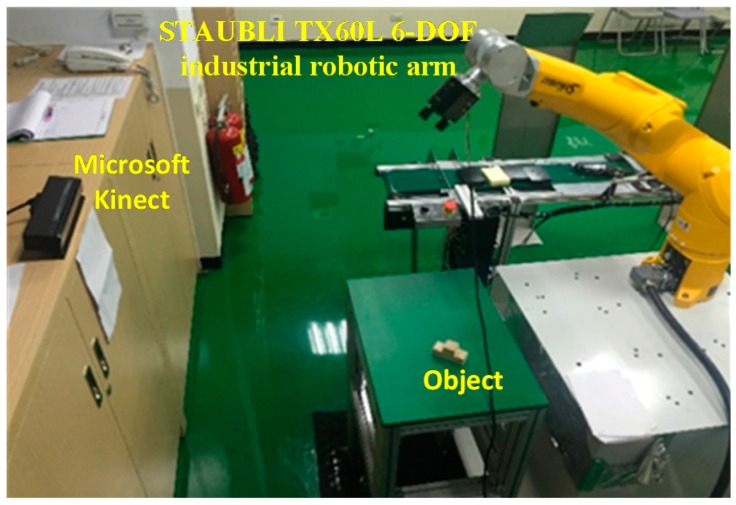
The experimental setup.

**Figure 15:**
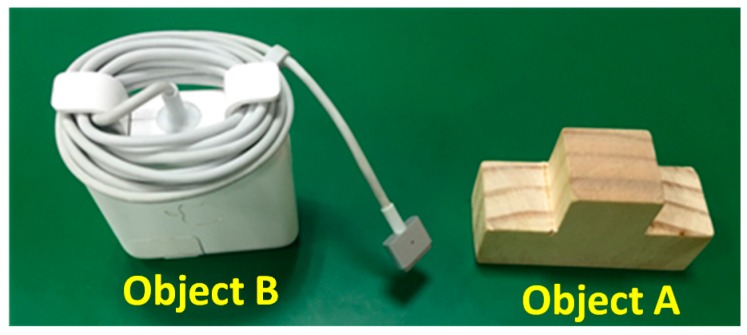
The work pieces used in the experiment.

Figure 14 shows the experimental steup. A TX60L (STAUBLI, Pfäffikon, Switzerland) 6-DOF industrial robotic arm was used to grasp objects and Microsoft Kinect v2 was used to capture the 3D point cloud data. The computer platform that was used for object recognition was equipped with an Intel i5 CPU, 8 GB DDRIII. The two objects that were randomly selected in the experiment are shown in Figure 15. Three and six stable poses were respectively established in a database for Objects A and B. The robotic arm was taught how to grasp the objects from each stable pose and the data for every 20° of rotation around the *z*-axis was then stored.

**Figure 16:**
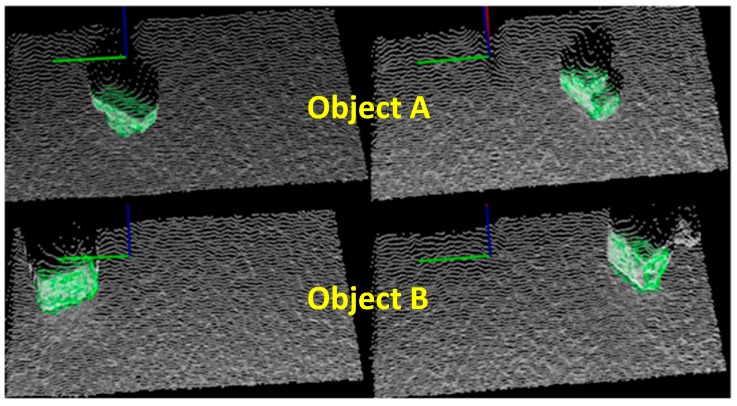
The results for object recognition.

**Figure 17:**
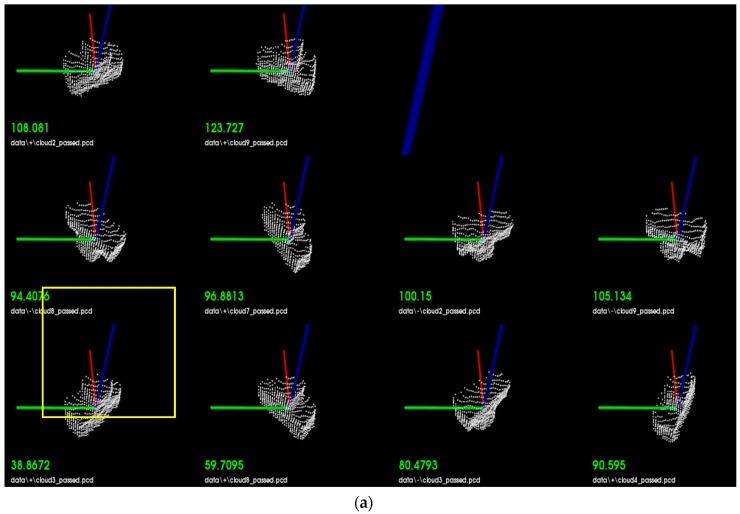

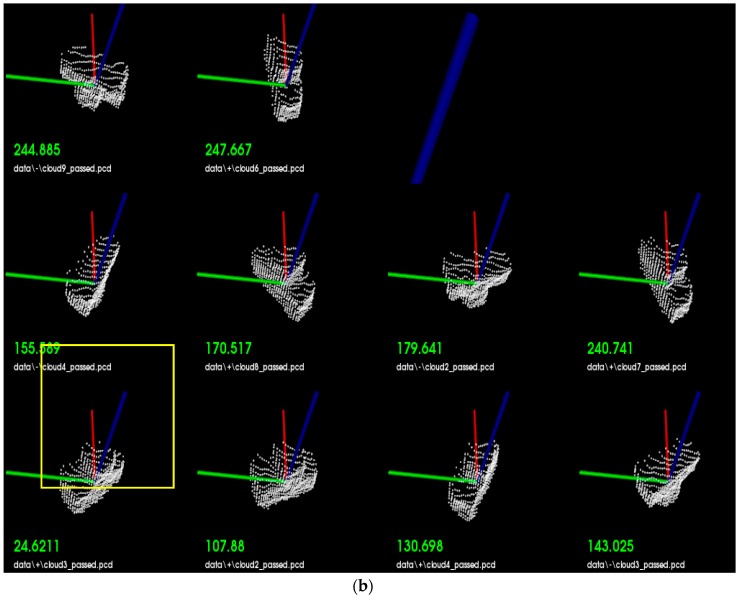
Analysis of the recognition capability: (**a**) using the VFH descriptor and (**b**) using the MVFH descriptor.

**Figure 18:**
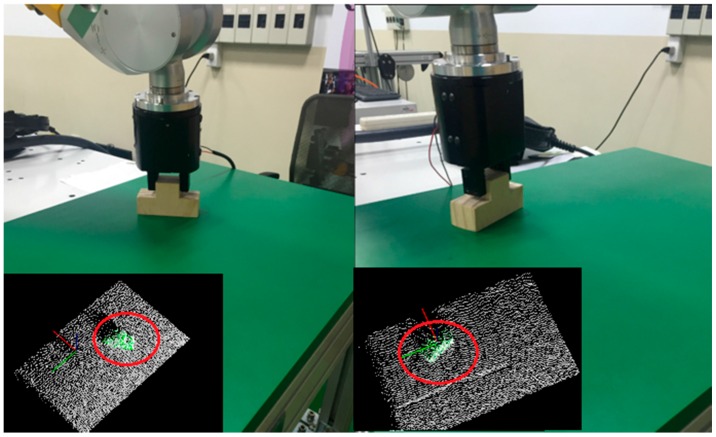
The reults for object grasping.

The objects were placed at random locations on the table. Figure 16 shows the results for object recognition and pose estimation. As can be seen from the Figure 16, the online scan (color: white) were closely aligned with the database for each stable position (color: green) To test the validity of the proposed MVFH descriptor, the first 10 matching scores (color: green) were used to determine the recognition capability, as shown in Figure 17. Figure 17a,b respectively show the results for object recognition using the VFH and MVFH descriptors. The yellow window indicates the optimal match. The scores for these two descriptors show that the proposed MVFH descriptor allows a greater recognition capability than the VFH descriptor. The results for the computation time that was required for the two objects are presented in Table 2. When the object was recognized and the corresponding rough pose was estimated using the VFH and the pose was further refined using ICP, the average computation time was 0.6019 s. The MVFH was also used, to avoid false pose recognition and the average time for computation was reduced to 0.4948 s because the refined pose converges faster using ICP. After object recognition and pose estimation, the refined pose was used to guide the robot to grasp the object. Figure 18 shows the results.

## 5. Conclusions

This study proposes a 3D object recognition and registration system for robotic grasping that uses a Kinect sensor. To ensure accurate pose estimation when an object is placed symmetrically in relation to the viewpoint, this study also proposes an MVFH descriptor that consists of two parts: a surface shape component that comprises an extended FPFH and an extended viewpoint direction component. The MVFH descriptor characterizes object poses and increases the system’s ability to identify objects with mirrored poses. The key contribution of this paper is the design of a new accurate and computationally efficient 3D feature that allows object recognition and pose identification for a VGR grasping system where there are 6-DOF. The experimental results show that the proposed VGR efficiently grasps different objects.

## Figures and Tables

**Table 1:** Computation time (unit: s).

Method	Average Computation Time
VFH	0.01691
With VFH + ICP	0.25634
MVFH	0.02162
With MVFH + ICP	0.22179

**Table 2:** Computation time (unit: s).

Method	Average Computation Time
With MVFH + ICP	0.4948
With VFH + ICP	0.6019
